# Genetically modified (GM) crop use 1996–2024: farm income and production impacts

**DOI:** 10.1080/21645698.2026.2726010

**Published:** 2026-09-06

**Authors:** Graham Brookes

**Affiliations:** PG Economics Ltd., Dorchester, UK

**Keywords:** Cost, genetically modified crops, income, production, yield

## Abstract

This paper updates previous estimates of the global value of using genetically modified (GM) crop technology in agriculture at the farm level. It examined impacts on yields, key variable costs, direct farm income and impacts on production of the main crops where the technology is used. The economic benefits have been significant with farm incomes for those using the technology having increased by $384.9 billion US dollars (1996–2024). This equates to an average farm income gain of $106/hectare. The farm income gains are divided 52% to farmers in developing countries and 48% to farmers in developed countries. Sixty-eight per cent of the gains have derived from yield and production gains with 32% coming from cost savings. These yield and production gains have made important contributions to increasing global production levels of the four main crops, having, for example, added 459 million tonnes and 817 million tonnes respectively to the global production of soybeans and maize since 1996. In 2024, the extra global production of the crops in which GM technology has been used (99 million tonnes), would have, if conventional production systems been used, required an additional 25.5 million ha of land to be planted to these crops. In terms of investment, for each extra dollar invested in GM crop seeds (relative to the cost of conventional seed), farmers gained an average US $4.17 in extra income. In developing countries, the average return was $5.31, and in developed countries the average return was $3.47.

## Introduction

Crops containing genetically modified (GM) traits have been widely grown since the mid-1990s, and in 2024 the global area harvested of these crops was about 205 million hectares. The main crops in which GM traits have been commercialized are soybeans, maize/corn, cotton and canola, with GM traits accounting for 48.5% of the global harvested area of these four crops in 2024. In terms of the share of the total global harvested area to these four crops, GM traits accounted for most soybean and cotton crops (73% and 75% respectively) with the GM shares being 32% for maize/corn and 25% for canola. The main GM traits used are insect resistant (IR) and herbicide tolerant (HT). In 2024, 58% of the GM crops harvested contained both HT and IR traits (commonly referred to as stacked traited seed), 34% contained only HT traits and 8% contained only IR traits.

The long-standing concentration of GM crop technology use in the two traits of herbicide tolerance and insect resistance in these four crops over the first quarter of a century of widespread global use likely reflects myriad reasons. Discussion of these reasons probably merits being the focus of a standalone paper and therefore is not examined in this paper. Nevertheless, the early dominance of these two categories of trait in four crops probably reflected the success that early GM crop technology developers had in demonstrating high levels of efficacy of the technology in controlling important production constraints in these four crops. These constraints included the development of pest resistance to insecticides commonly used to control major pests in crops like cotton, important pests such as stem borers in maize being difficult to control with insecticides and several weeds that compete with crops like soybeans becoming resistant to selective herbicides traditionally applied for control. The continued concentration of the two main categories of trait and four crops also recognizes the popularity of, and familiarity with the technology that has occurred among farmers of these crops so that as newer, improved second and third generations of the technology have become available they have been willing to adopt and rapidly scale up usage.

Since the introduction of GM crop technology, there have been many analytical papers assessing the farm level economic and income impacts associated with the adoption of this technology. The author of this paper has undertaken some of these studies (e.g., Brookes)^1^ and, since 2005, has engaged in a regular exercise to identify, update and aggregate the sum of these various studies, and where possible, to supplement them with new analysis. The aim of this has been to provide an up to date and as accurate as possible assessment of some of the key farm level economic impacts associated with the global adoption of crops containing GM traits. It is also hoped the analysis continues to contribute to understanding the impact of this technology and to facilitate more informed decision-making, especially in countries where crop biotechnology is currently not permitted.

This study updates the findings of earlier analysis into the global impact of GM crops since their commercial introduction in 1996 by extending analysis to include the years of 2021–2024. Previous analysis by the current author has been published in various journals, with the last analysis being Brookes^2^. The methodology and analytical procedures in this present discussion are unchanged. This allows a direct comparison of the new with earlier data and analysis. Readers should note that some data presented in this paper are not directly comparable with data presented in previous analysis because the current paper also includes new data and analysis that may have not previously been available, including revisions to some data for earlier years.

To save readers of this paper, the chore of consulting the past papers for details of the methodology and arguments, these are included in full in this paper.

The analysis focuses on gross farm income effects because these are a primary driver of adoption among farmers (both large commercial and small-scale subsistence). It also quantifies the (net) production impact of the technology. The author recognizes that an economic assessment could examine a broader range of potential impacts (e.g., on labor usage, household incomes, local communities and economies). However, these are not included because undertaking such an exercise would add considerably to the length of the paper, and an assessment of wider economic impacts would probably merit a separate assessment.

## Methodology

The report is based on a detailed analysis of existing farm level impact data for GM crops, much of which can be found in peer reviewed literature. Most of this literature broadly refers to itself as “economic impact” literature and applies farm accounting or partial budget approaches to assess the impact of GM crop technology on revenue, the main variable costs of production (seed cost, crop protection and weed control, use of labor and fuel/machinery) and gross farm income. Although primary data relating to impacts of commercial cultivation of crops containing GM traits were not available for every crop, in every year and for each country, a substantial body of representative research and analysis is available, and this has been used as the main basis for the analysis presented. The author has also undertaken his own analysis of the impact of some trait-crop combinations in some countries where the availability of published research is more limited (notably GM herbicide tolerant [HT] traits). This is based on analysis of key input use data such as seed, herbicide and insecticide usage and costs.

The farm level economic impact of the technology varies widely, both between and within regions/countries and by year. Therefore, the analysis is considered on a case-by-case basis, using average performance and impact recorded in different crop and trait combinations by the studies reviewed. Where more than one piece of relevant research (e.g., on the impact of using a GM trait on the yield of a crop in one country in a particular year) has been identified, the findings used in this analysis reflect the authors assessment of which research is more likely to be reasonably representative of impact in the country as a whole and in a particular year. For example, there are many papers on the impact of GM insect resistant (IR) cotton in India in its early years of widespread usage. Few of these studies were reasonably representative of cotton growing across the country, with most based on small-scale, local and therefore unrepresentative samples of cotton farmers. Only the findings from research drawn from reasonably representative samples of farms using the technology and growing the crops have been drawn on for use in this paper – readers should consult the references section to identify the sources used.

This approach may still overstate, or understate, the impact of GM technology for some trait, crop and country combinations, especially in cases where the technology has provided yield enhancements. However, as impact data for every trait, crop, location and year is not available, extrapolation from available studies to years for which no data are available has been required. In addition, some studies assessed impact in the early years of adoption and extrapolation from such a base may not adequately reflect the nature of recent or currently available alternative (non-GM seed or crop protection) technology. The author acknowledges that these factors represent potential methodological weaknesses. To reduce the possibilities of over/understating impact due to these factors, the analysis:

- Directly applies impacts identified from the literature to the years that have been studied. As a result, the impacts used vary in many cases according to the findings of literature covering different years. Examples where such data is available include the impact of GM insect resistant (IR) cotton: in India (see Bennett R et al.,^3^ IMRB^4^ and IMRB^5^), in Mexico (see Traxler et al.^6^ and Monsanto/Bayer Mexico annual monitoring reports submitted to the Ministry of Agriculture in Mexico^7^), in the USA (see Sankala & Blumenthal^8^^,^^9^), Mullins & Hudson^10^) and in China (see Quan Y et al.^11^). Hence, the analysis considers variation in the impact of the technology on yield according to its effectiveness in dealing with (annual) fluctuations in pest and weed infestation levels;
- Uses current farm level crop prices and bases any yield impacts on (adjusted – see below) current average yields for each year. This provides a degree of dynamic analysis that would, otherwise, be missing if constant prices and average yields identified in year-specific studies had been used;
- Includes changes and updates to the impact assumptions identified in the literature based on new papers, annual consultation with local sources (analysts, industry representatives, databases of crop protection usage and prices) and analysis of changes in crop protection product usage and prices and of seed varieties planted and their prices;
- Adjusts downwards the average base yield (in cases where GM technology has been identified as having delivered yield improvements) on which the yield enhancement has been applied. In this way, the impact on total production is not overstated.

Detailed examples of how the methodology has been applied to calculate the 2024 impacts are presented in Appendix A.

Other aspects of the methodology used to estimate the impact on direct farm income are as follows:

- Where stacked traits have been used, the individual trait components were analyzed separately to ensure estimates of all traits were calculated. Even though stacked traited seed accounts for most of all GM crops grown in recent years this is possible because the non-stacked seed has been (and in some cases continues to be) available and used by farmers and there are studies that have assessed these trait-specific impacts;
- All values presented are nominal for the year shown and the base currency used is the US dollar. All financial impacts in other currencies have been converted to US dollars at prevailing annual average exchange rates for each year (source: United States Department of Agriculture Economics Research Service);
- The analysis focuses on changes in farm income in each year arising from the impact of GM technology on yields and the key costs of production, seed cost and crop protection expenditure. It also considers impact on costs such as fuel and labor usage. Inclusion of these latter costs is more limited than the impacts on seed and crop protection costs because only a few of the papers reviewed have included consideration of such costs. Therefore, in most cases the analysis relates to impact of crop protection and seed cost only, crop quality (e.g., improvements in quality arising from less pest damage or lower levels of weed impurities which result in price premia being obtained from buyers) and the scope for facilitating the planting of a second crop in a season (e.g., second crop soybeans in Argentina following wheat that would, in the absence of the GM HT seed, probably not have been planted). The farm income effect presented is essentially a gross margin impact (gross revenue minus variable costs of production) rather than a full net cost of production assessment. Through the inclusion of yield impacts and the application of actual (average) farm prices for each year, the analysis also indirectly considers possible impacts of GM crop adoption on global crop supply and world prices.

The paper also includes estimates of the production impacts of GM technology at the crop level. These have been aggregated to provide the reader with a global perspective of the broader production impact of the technology. These impacts derive from the yield impacts and the facilitation of additional soybean cropping within a season in South America. Details of how these values were calculated (for 2024) are shown in Appendix 1.

## Results and Discussion

### Herbicide Tolerant (HT) Crops

GM HT crops were first grown widely in 1996, and, in 2024, 92% of all the GM crops harvested used HT traits either via stacked traited seed (with IR traits) or HT only. In the first 20 y of usage, most GM HT crops were solely tolerant to the herbicide active ingredient glyphosate, although since about 2015, the availability and use of crops tolerant to other herbicides has increased. The main impact of this technology has been to provide more cost effective (less expensive) and easier weed control for farmers. Some users of this technology have also obtained higher yields from better weed control (relative to weed control obtained from conventional technology). The magnitude of these impacts varies by country and year, and the variation is due to several factors. These include the prevailing costs of different herbicides used in GM HT systems versus weed control practices in conventional (non-GM crops), which may include different/alternative herbicides to those used with GM HT crops and/or other forms of weed control (e.g., hand or mechanical weeding), the mix and amounts of herbicides applied, the cost farmers pay for accessing the GM HT technology and the underlying levels of weed problems faced by farmers. Important factors affecting the level of cost savings achieved include:

- The mix and amounts of herbicides used on GM HT crops and conventional crops are affected by price and availability of herbicides. Herbicides used include both “older” products that are no longer protected by patents and newer “patent-protected” chemistry, with availability affected by commercial decisions of suppliers to market or withdraw products from markets and regulation (e.g., changes to approval processes and the imposition of restrictions/bans). Prices also vary by year and country according to factors such as exchange rates, costs of manufacture and distribution;
- The amount farmers pay for use of the technology varies by country and year. Pricing of technology (all forms of seed and crop protection technology, not just GM technology) varies according to the level of benefit that the technology providers perceive farmers are likely to derive from it. In addition, it is influenced by patent protection, plant breeders’ rights and rules relating to use of farm-saved seed. In countries with weaker intellectual property rights, the cost of the technology tends to be lower than in countries where there are stronger protected rights. This issue is examined further below as it is a key factor determining take up levels of the technology. Also, the HT technology available in recent years is commonly not the same as the technology available in the early years of adoption. As indicated above, in the first 15–20 y of widespread use of GM HT crop technology, crops tolerant to glyphosate dominated. In the last 10-y, farmers notably in North and South America now have the option of using seed tolerant to glyphosate plus other herbicide active ingredients like glufosinate, 2,4-D and dicamba. These forms of “stacked” herbicide tolerances are typically more expensive than the single herbicide tolerance traits of the early years of use;
- Where GM HT crops tolerant to glyphosate have been widely grown for a number of years, incidence of weed resistance to glyphosate has increased and become a major concern in many regions. This has been attributed to how glyphosate was used with GM HT crops in the early years of adoption. Due to its broad-spectrum, post-emergence activity and effectiveness in controlling weeds cheaply, it was often used as the sole method of weed control. This approach to weed control put tremendous selection pressure on weeds and contributed to the evolution of weed populations predominated by resistant individual weeds. It should, however, be noted that there are hundreds of resistant weed species confirmed in the International Survey of Herbicide Resistant Weeds (www.weedscience.com).^12^ Worldwide, there are 62 weed species that are currently resistant to glyphosate (accessed May 2026), compared to 176 weed species resistant to ALS herbicides (e.g., chlorimuron ethyl commonly used in conventional soybean crops) and 89 weed species resistant to photosystem II inhibitor herbicides (e.g., atrazine commonly used in maize production). It should also be noted that the problem of herbicide‑resistant weeds has not been accelerated or exacerbated by the adoption of GM HT crops and the overall rate of newly confirmed herbicide-resistant weed species to all herbicide sites of action has slowed in the US since 2005 (Kniss^13^). In addition, GM HT technology has played a major role in facilitating the adoption of no and reduced tillage production techniques in North and South America. This has also probably contributed to the emergence of weeds resistant to glyphosate and to weed shifts toward those weed species that are less well controlled by glyphosate. As a result, growers of GM HT crops have increasingly been (and continue to be) advised to include other herbicides, with different and complementary modes of action, in combination with glyphosate in their weed management systems, even where instances of weed resistance to glyphosate may have not been found. In some cases, farmers may also be advised to supplement with cultural weed control practices such as plowing. This change in weed control practices also reflects the broader agenda of developing strategies across all forms of cropping systems to minimize and slow the potential for weeds developing resistance to existing weed control technology (e.g., Norsworthy et al,^14^). In addition, in the last 10 y, the increasing array of new GM HT technology referred to above has offered farmers (notably in North and South America) crops that are tolerant to other herbicide active ingredients typically in combination with tolerance to glyphosate (and sometimes offering tolerance to three active ingredients). At the macro level, these changes have influenced the mix, total amount, cost and overall profile of herbicides applied to GM HT crops. It has also resulted in the weed control costs associated with growing GM HT crops generally being higher in the last 10 y than in the early 2000s. However, as the analysis presented below shows, GM HT crops have continued to be popular with farmers as they offer important economic advantages for most users relative to the conventional (non-GM) alternative, either in the form of lower costs of production or higher yields (arising from better weed control). An important contributory factor to this (maintenance of cost saving advantage of GM HT systems versus conventional alternatives) is that many of the herbicides used in conventional production systems also face significant weed resistance issues themselves (in the mid‑1990s this was one of the reasons why glyphosate tolerant soybeans were rapidly adopted, as glyphosate provided good control of these weeds). It is also important to note that if GM HT technology was no longer delivering net economic benefits, it is likely that farmers around the world would have significantly reduced their adoption of this technology in favor of conventional alternatives. The fact that GM HT global crop adoption levels have not fallen in recent years suggests that farmers must be continuing to derive important economic benefits from using the technology.

These points are further illustrated in the analysis below.

### GM HT Soybeans

The most common farm income gain arising from the use of this seed technology has derived from a reduction in the cost of production, mainly through lower expenditure on weed control (typically herbicides). These gains have averaged between $6/ha and $34 ha (Table 1).

**Table 1.** GM HT soybeans: summary of average gross farm level income impacts 1996–2024 ($/hectare).

Country	Due to cost savings	Due to higher yields	Due to facilitation of second cropping
Romania	9	35.6	Not applicable
Argentina	22.5	Not applicable	211
Brazil	33.9	Not applicable	Not applicable
USA	33.5	113.3	Not applicable
Canada	21.0	123.4	Not applicable
Paraguay	16.7	Not applicable	150
Uruguay	23.9	Not applicable	Not applicable
South Africa	7.2	Not applicable	Not applicable
Mexico	12.2	27.8	Not applicable
Bolivia	6.0	77.2	Not applicable

1. Romania applies to 1999 to 2006 only. Mexico 2004–2016 only 2. Higher yield impact for USA and Canada relates to higher yielding second generation GM HT soybeans from 2008 3. All values presented for cost savings are net after deduction of the cost of the technology. For further information see Appendix B.

Where yield gains have occurred, from improvements in weed control, the average farm income gain has been higher, for example in countries such as Romania, Mexico and Bolivia, where additional income gains of between $28/ha and $77/ha have been obtained. A second generation of GM HT soybeans also became available to commercial soybean growers in the US and Canada in 2009 which offered the same tolerance to glyphosate as the first generation of the GM HT seed (and the same cost saving) but with higher yielding potential. The realization of this potential is shown in the higher average gross farm income benefits (Table 1). GM HT soybeans have also facilitated the adoption of no and reduced tillage production systems in some countries, shortening the production cycle by reducing the time required for harvesting and drilling subsequent crops. This advantage has enabled many farmers in South America to plant a crop of soybeans immediately after a wheat crop in the same growing season. The second crop, additional to traditional “one crop” soybean production, has therefore added considerably to farm incomes and to the volumes of soybean production in countries such as Argentina and Paraguay (Table 1).

In global terms, the farm level impact of using GM HT technology in soybeans (excluding “Intacta soybeans” which have been grown widely in South America since 2013 and combine GM herbicide tolerance with GM insect resistance traits in soybeans: see below) was $5.25 billion in 2024. If the second crop benefits arising in Argentina and Paraguay are included, the total is $6.63 billion. Cumulatively since 1996, the farm income benefit has been (in nominal terms) $81.2 billion ($103.3 billion if second crop gains in Argentina and Paraguay are included).

In terms of the total value of global soybean production in 2024, the additional farm income (inclusive of Argentine second crop gains) generated by the technology is equal to a value-added equivalent of 4%. These economic benefits should be placed within the context of a significant increase in the level of soybean production in the main GM adopting countries since 1996 (more than a doubling in the area planted in the leading soybean producing countries of the US, Brazil and Argentina).

If it is assumed that the second crop soybean gains are effectively *de facto* “yield” gains then of the total cumulative farm income gains from using GM HT soybeans, $68.8 billion (67%) is due to yield gains/second crop benefits and the balance, 33%, is due to cost savings. The important contribution of these second crop production gains (plus yield enhancements) to global supplies of soybeans is discussed further below (crop production impacts).

### GM HT and IR (Intacta) Soybeans

This combination of GM herbicide tolerance (to glyphosate) and insect resistance in soybeans was first grown commercially in 2013 in South America. Since then, the technology has been used on 343 million hectares in Argentina, Brazil, Paraguay and Uruguay and contributed an additional $39.9 billion to gross farm income of soybean farmers in these countries. Brazil accounted for 78% of the area planted to this seed and 82% of the total farm income gain associated with its use.

**Figure 1.**
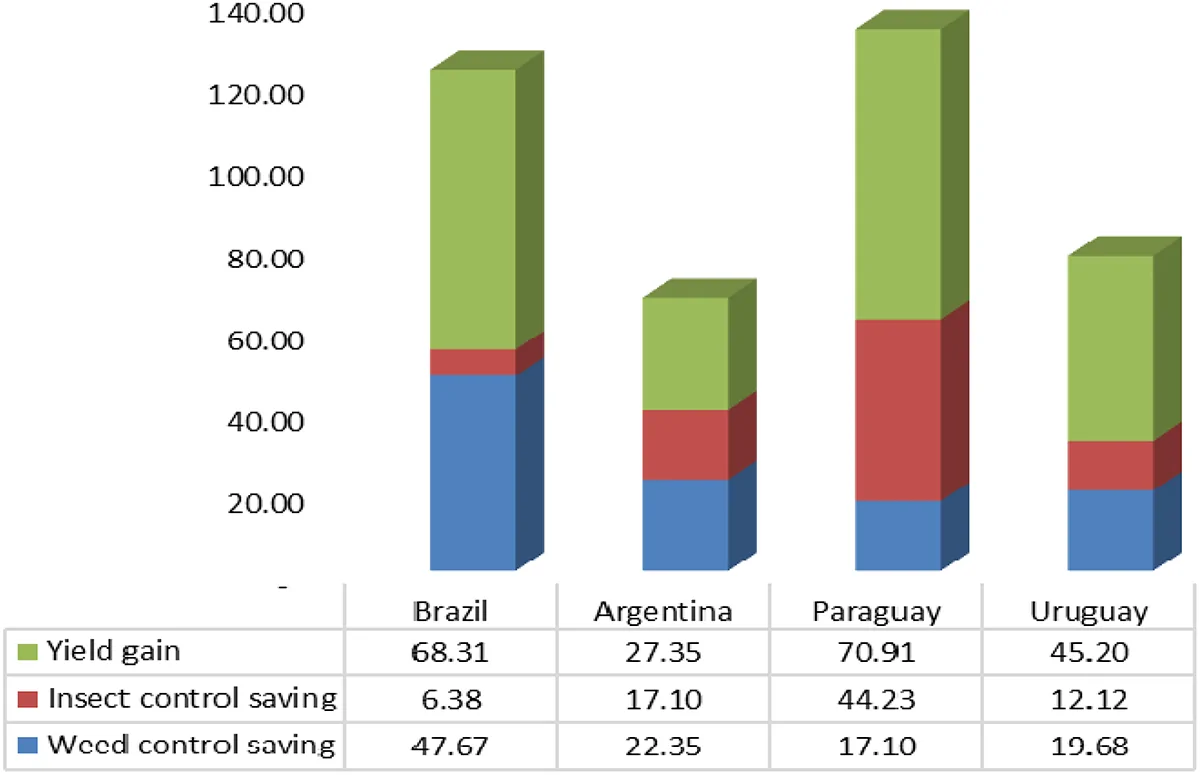
Breakdown of sources of income gain with Intacta soybeans by country 2013–2024 ($/ha).

The average income gains over the 8 y of adoption have been respectively $122.4/ha, $66.8/ha, $132.2/ha and $77/ha in Brazil, Argentina, Paraguay and Uruguay, through a combination of cost savings (decreased expenditure on herbicides and insecticides) and higher yields (see Figure 1).

Additional details of the income gain components are available in Appendix B. All values presented for cost savings are net after deduction of the cost of the technology.

### GM HT Maize

**Figure 2.**
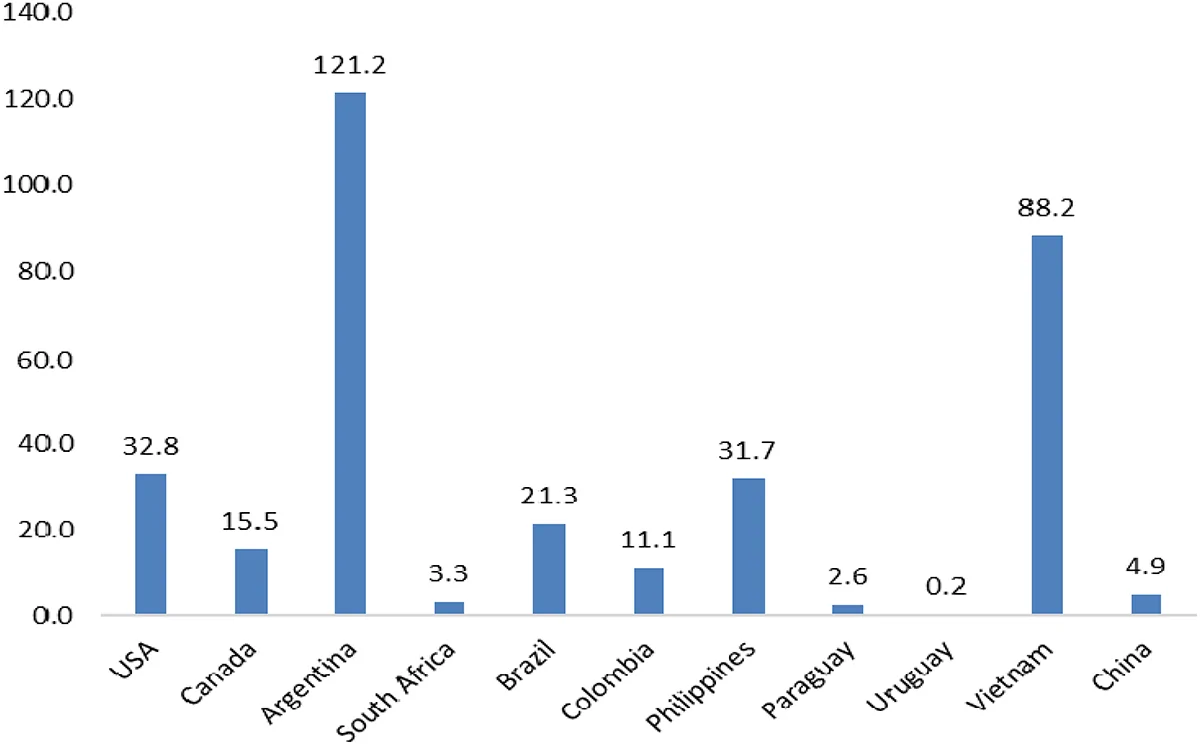
Average farm income gain from using GM HT maize by country: 1997–2024 ($/ha).

The adoption of GM HT maize has mainly resulted in lower costs of production, although yield gains from improved weed control have arisen in Argentina, Brazil, the Philippines and Vietnam. As a result, the average level of income gain where cost savings have been the sole form of income gain has varied between $0.2/ha in Uruguay to nearly $33/ha in the USA, with the highest gains being in countries where cost savings combined with yield gains derived from improved weed control (notably Argentina and Vietnam: Figure 2).

Additional information relating these gains can be found in Appendices 1 and 2. All values presented are net after deduction of the cost of the technology.

In 2024, the total global farm income gain from using this technology was $2.45 billion with a cumulative gain over the period 1997–2024 of $31.5 billion. Within this, $14.5 billion (46%) was due to yield gains and the rest derived from lower costs of production.

### GM HT Cotton

A similar pattern of impact on farm income has occurred with the adoption of GM HT cotton since 1997. Most farmers have obtained cost savings, with some (in South America and Mexico) obtaining yield gains from improved levels of weed control. In 2024, the use of this technology delivered a gross farm income gain of $462.2 million, and, in the 1997–2024 period, the total gross farm income benefit was $4.45 billion. Fifty-seven per cent of these gains have been derived from cost savings, with the remaining 43% from yield gains.

Additional details relating to the nature of these income gain calculations in each adopting country are presented in Appendices 1 and 2.

### Other HT Crops

**Figure 3.**
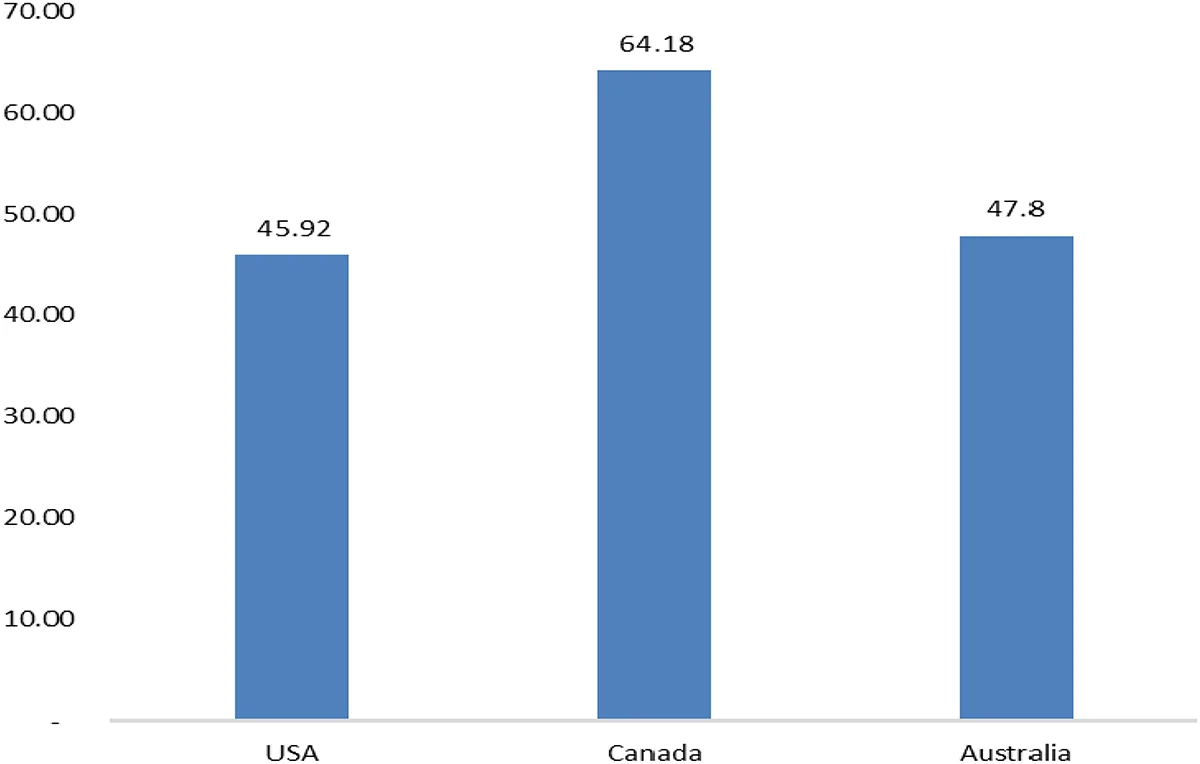
Average farm income gain from using GM HT canola by country: 1996–2024 ($/ha).

GM HT canola (tolerant to glyphosate or glufosinate) has been grown in Canada since 1996, in the USA since 1999 and in some states of Australia since 2008. The farm income impact in all three countries has been a combination of yield gains and some cost of production (weed control) savings, with the average income gain being within a range of $46/ha in USA to $64/ha in Canada (Figure 3). In 2024, the total global income gain from the adoption of GM HT technology in canola was $650.7 million, and cumulatively since 1996 it has been $11.6 billion. Within this, 78% has been due to yield gains, and the balance (22%) has been from cost savings. In terms of the total value of canola production in these three countries in 2024, the additional farm income generated by the technology is equal to a value-added equivalent of 5.3%.

Additional information relating these gains can be found in Appendices 1 and 2. All values presented are net after deduction of the cost of the technology.

GM HT (tolerant to glyphosate) sugar beet has been grown in the USA and Canada since 2008. The impact of using this technology has been to deliver a combination of yield gains and reductions in the cost of production (weed control). Since 2008, the average farm income gain has been $146/ha, of which 88% has derived from weed control cost savings and the balance of 12% has come from higher yields. In 2024, the total farm income benefit from using GM HT sugar beet in the USA and Canada was $101.4 million, and since 2008 the cumulative income gain has been $1.12 billion. Additional information relating to these farm income gains are presented in Appendices 1 and 2.

### Insect Resistant (GM IR) Crops

GM IR crops were also first grown widely in 1996 but only in maize and cotton. From 2000, stacked traited maize and cotton seed (stacking of IR and HT traits) became available to farmers in North America and within 10 y become the primary type of GM seed used by farmers of maize and cotton in North (and latterly South) America. As a result, by 2024 more than 90% of all global GM maize harvested derived from stacked traited maize, with less than 10% of GM IR maize use being from seed containing only IR traits. Similarly, IR traited cotton is commonly stacked with HT traits in the cotton crops of countries in the Americas, though in Asia (notably China and India), IR traited only cotton continues to be the only form of GM cotton grown. As China and India accounted for 63% of the total GM cotton area harvested in 2024, GM IR only traited cotton accounted for just over 70% of the total GM cotton area. GM IR soybeans became available in 2013 to farmers in Argentina, Brazil, Paraguay and Uruguay stacked with HT traits and by 2024 accounted for just under 50% of the global GM soybean harvested area.

In the early years of usage, most GM IR crops operated via a single mode of action against a small range of pests (e.g., corn boring pests in maize and bollworm pests in cotton) before second and latterly third generation traits became available that offer pest protection via more modes of action and contain protection against additional pests (e.g., Fall Armyworm in maize).

The main way in which GM IR technologies have impacted on farm incomes has been through higher yields derived from reduced levels of pest damage. Many users of this technology also obtained cost savings from reduced use of insecticides with the traits effectively replacing the insecticides previously used to control some pests. As with GM HT traits, the magnitude of these impacts varies by country and year, and the variation is due to several factors. These include the prevailing costs of the GM IR traited seed, the use and cost of insecticides still used in GM IR crops to control pests not controlled by the traits versus the cost of non-GM IR seed and pest control practices in conventional (non-GM crops) plus the underlying levels of pest pressure faced by farmers.

The same factors that impact the mix and amounts of herbicides used on GM HT crops and conventional crops apply to the use and mix of GM IR crops. These include:

- The amount farmers pay for use of the technology varies by country and year. Pricing of IR seed technology (and alternative forms of pest control) varies according to the level of benefit that the technology providers perceive farmers are likely to derive from it. In addition, the weaker provision of patent protection, plant breeders’ rights and rules relating to use of farm-saved seed in developing countries relative to developed countries has resulted in lower average cost of the technology in developing countries;
- The IR technology available in recent years is commonly not the same as the technology available in the early years of adoption. In the early years of usage, most GM IR crops operated via a single mode of action against a small range of pests before second and latterly third‑generation traits became available that offer pest protection via more modes of action and contain protection against additional pests. These latter generations of IR traits with more than one mode of action and targeting a wider range of pests are commonly more expensive than the early generations of IR traits;
- Where GM IR crops have been widely grown for many years, some incidence of pest resistance to the IR traits have occurred (see for example, Tabashnik and Carrière,^15^). As a result, growers in regions affected have supplemented or rotated the use of GM IR traited seed with the use of other forms of pest control. In addition, the increasing availability of new IR traited seed with multiple modes of action to control pests have been used. These changes have resulted in the costs associated with growing GM IR crops generally being higher in the last 10 y than in the early years of adoption. The continued popularity of GM IR traited crops with farmers in 2024 does, nevertheless suggest that they continue to provide economic advantages for most users relative to the conventional (non-GM) alternative. The evidence relating to this is summarized below.

### GM IR Maize

**Figure 4.**
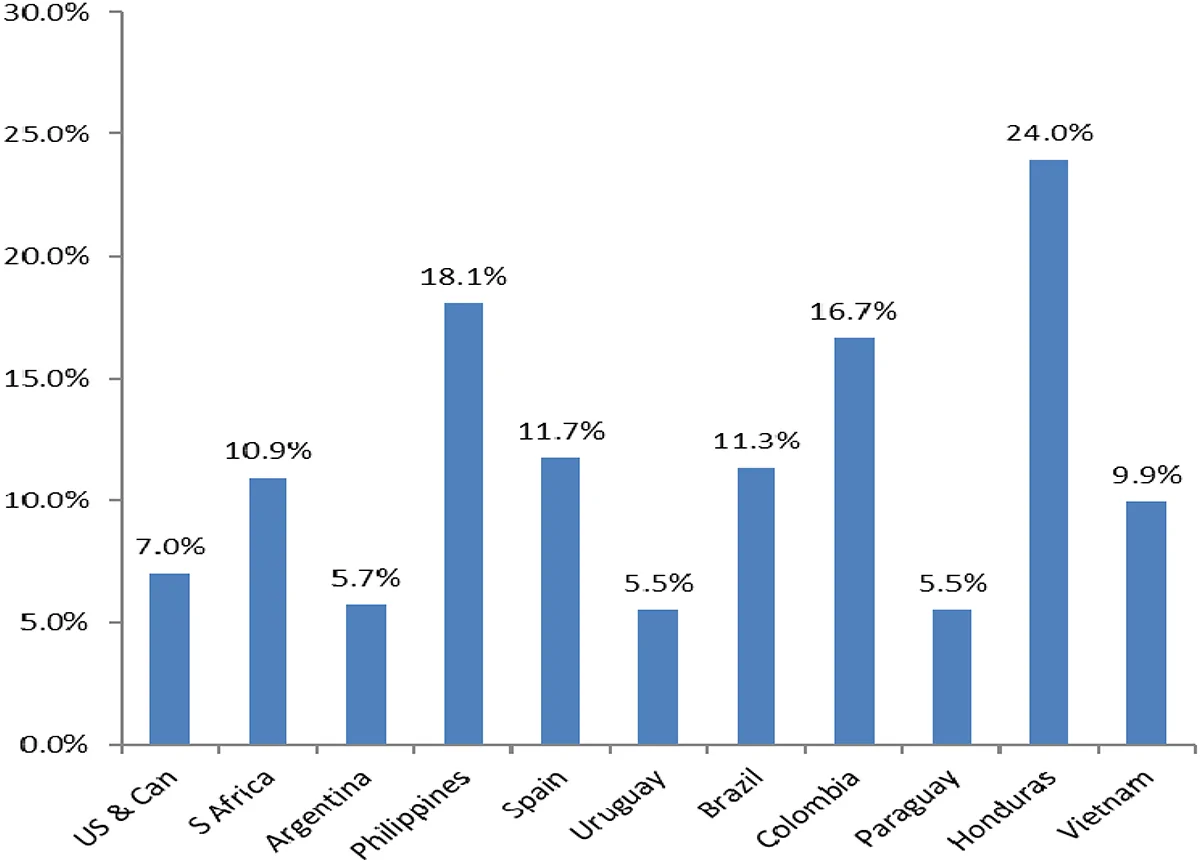
Average yield gains GM IR maize by country 1996–2024.

This technology targets various stalk boring pests that can cause significant yield losses to farmers around the world. In addition, in North America many GM varieties also contain traits that target the corn rootworm pest. The average yield gain performance of this technology since it was first used in 1996 is shown in Figure 4. Positive yield gains in the range of +5% to +24% have been recorded with an average yield gain across all user countries of +10.2%. The highest yield gains have occurred in developing countries, where conventional methods of pest control tend to be least effective (e.g., reasons such as poorly developed extension and advisory services, lack of access to finance to fund use of crop protection application equipment and products).

Additional information relating these gains can be found in Appendices 1 and 2.

**Figure 5.**
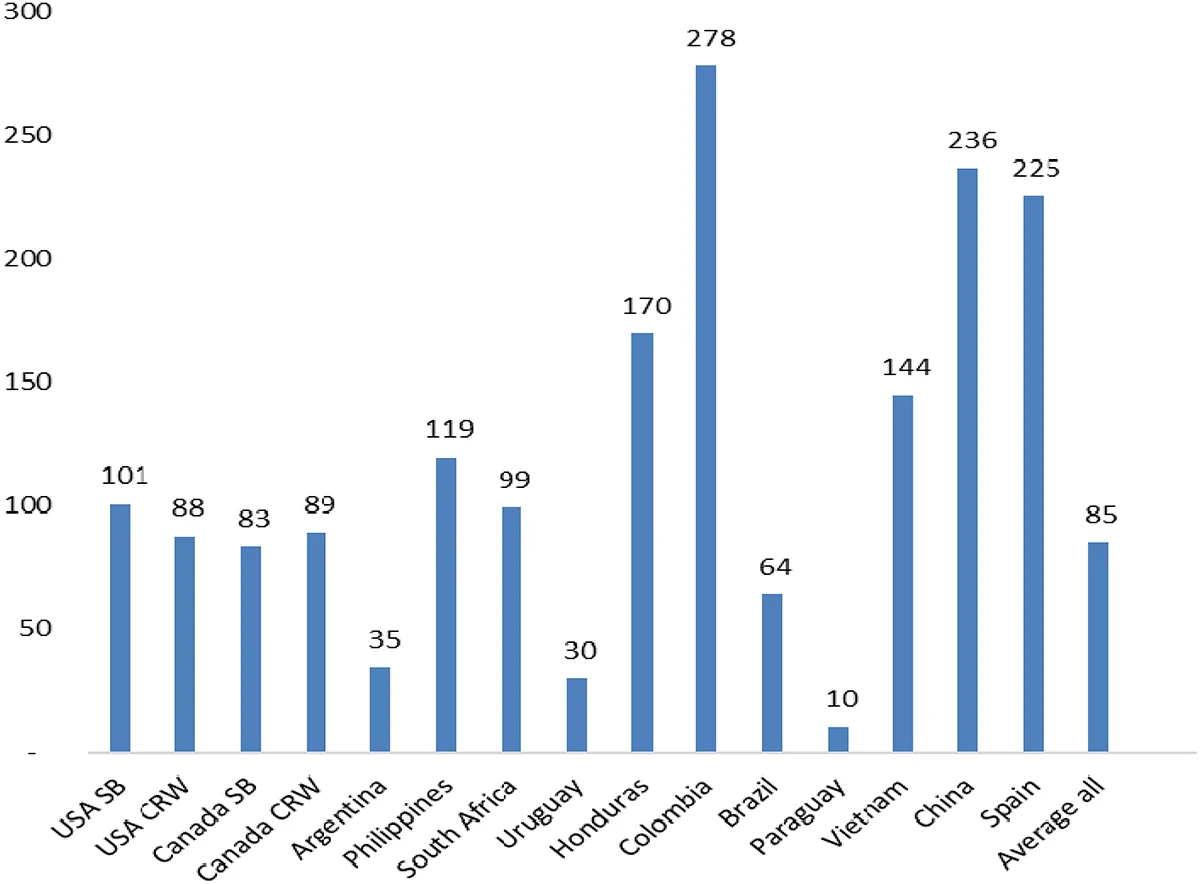
Average farm income gains GM IR maize by country 1996–2024 ($/ha).

The impact of these yield gains, coupled with some cost savings associated with less expenditure on insecticides and crop pest monitoring, on farm incomes is summarized in Figure 5. This shows that the average increase in farm income from using GM IR maize technology over the 1996–2024 period has been +$85/ha, within a range of +$10/ha and +$278/ha by user country. Aggregating these farm income gains, the total increase in farm income due to the use of GM IR maize between 1996 and 2024 has been $103.9 billion, with the increase in income in 2024 having been $5.4 billion.

Additional information relating these gains can be found in Appendices 1 and 2. All values presented are net after deduction of the cost of the technology. SB = targeting stem boring and earworm pests, CRW = targeting corn rootworm.

### GM IR Cotton

**Figure 6.**
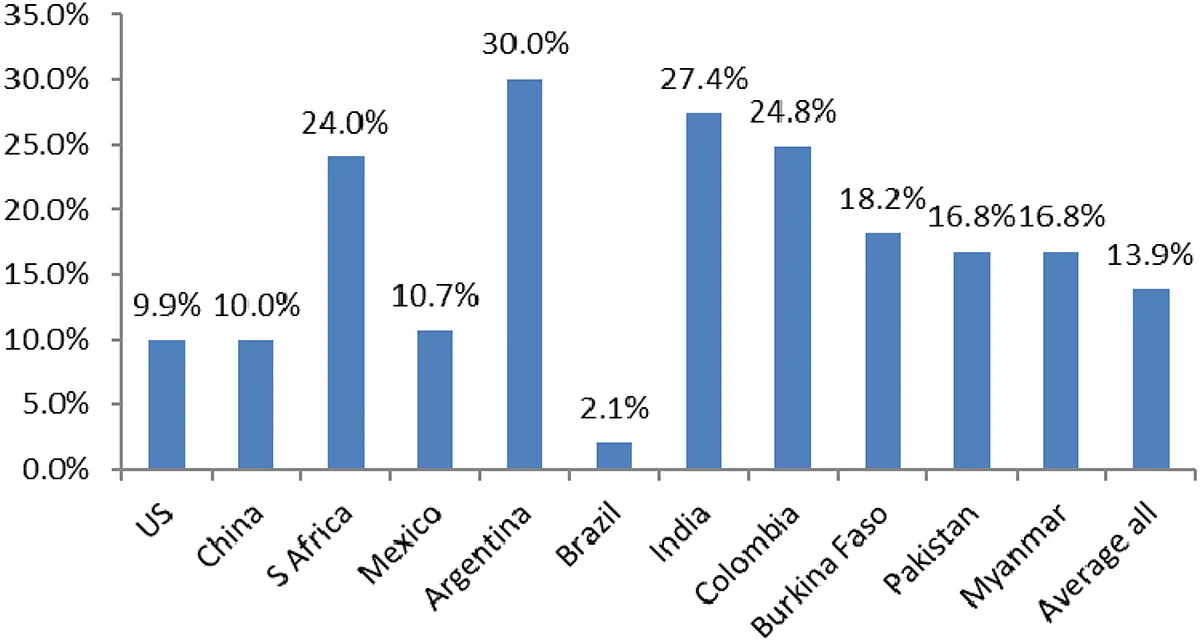
Average yield gains GM IR cotton by country 1996–2024.

GM IR cotton seed technology helps farmers control the various bollworm/budworm pests that can cause major problems for most cotton farmers. Before the availability of this technology, cotton crops in some countries (e.g., China) were routinely sprayed 15–20 times per year to control these pests. With availability of GM IR seed technology, the number and frequency of insecticide applications have fallen significantly to, typically less than five, and focused on control of pests that the GM IR technology does not control (e.g., sucking pests). The primary benefit of using this technology has been higher yields, with the average improvement in yield across all user countries between 1996 and 2024 having been +13.9%. Yield gains have been highest in developing countries (Figure 6). In addition, most farmers have gained from reduced costs of production via notable reductions in the amount and frequency of insecticide applications. The only adopting country that has not experienced a yield improvement from using GM IR cotton technology has been Australia, where the levels of boll and bud worm pests were relatively low before the first availability of GM IR technology because of effective use of intensive insecticide use programs. The main benefit of this technology in Australia has been the significant cost savings and the associated environmental gains from reduced insecticide use.

Additional information relating these gains can be found in Appendices 1 and 2.

**Figure 7.**
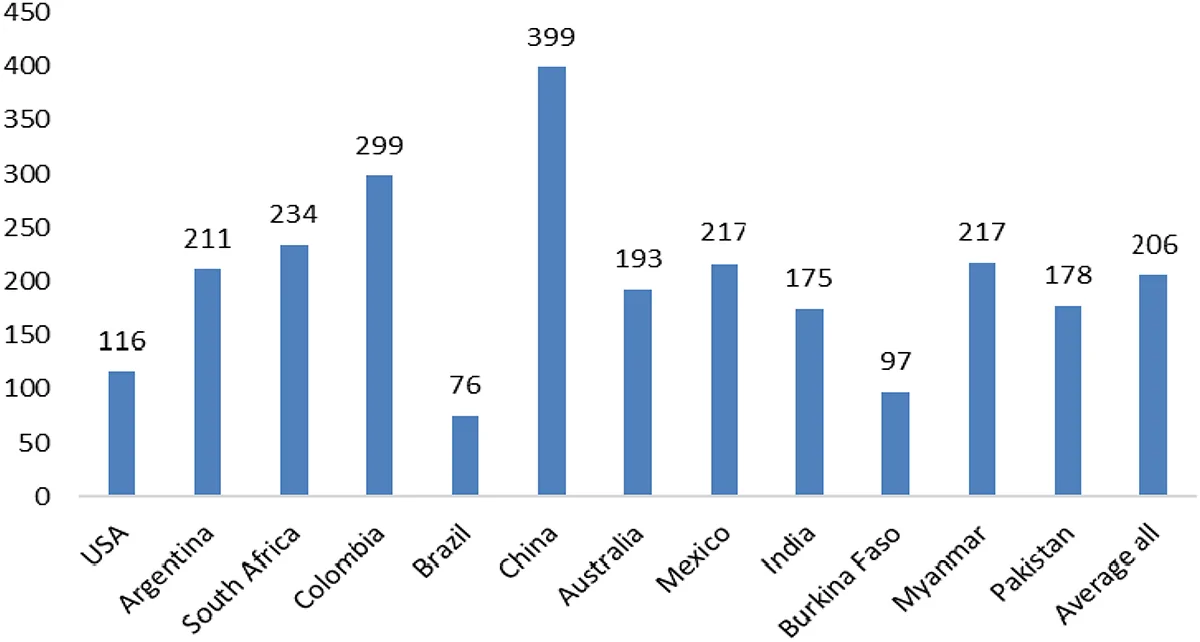
Average farm income gains GM IR cotton by country 1996–2024 ($/ha).

The average farm income gain from using GM IR cotton technology during the period 1996–2024 has been +$206/ha, with the highest levels of increase having been recorded in developing countries like China and Colombia (Figure 7).

Additional information relating these gains can be found in Appendices 1 and 2. All values presented are net after deduction of the cost of the technology.

At the aggregate level, the global gross farm income gains from using GM IR cotton in 2024 was $3.9 billion, and cumulatively since 1996 the gains have been $88.3 billion. Within this, 86% of the farm income gain has derived from yield gains (less pest damage) and the balance (14%) from reduced expenditure on crop protection (spraying of insecticides).

### GM Drought Tolerant Maize

GM drought tolerant maize has been growing in parts of the US since 2014 and in 2024 was planted on 1 million hectares. Drawing on yield comparison data with other drought tolerant maize (varieties conveying drought tolerance that is not derived from GM technology) from field trials (source: Monsanto US Field Trials Network in the Western Great Plains^16^), this suggests that the technology is providing users with a net yield gain of about 2.6% and a small cost saving in irrigation costs. After taking into consideration, the additional cost of the seed compared to non-GM drought tolerant maize, the average gross farm income gain (2014–2024) has been $33.6/ha. In 2024, this resulted in an aggregate farm income gain of $66.2 million and over the period 2014–2024, a total gain of $417.6 million.

### Aggregated (Global Level) Farm Income Impacts

GM crop technology has been used widely by many farmers for nearly 30 y. It has helped farmers adapt their weed and pest control practices and enabled important improvements in yields to be realized. In turn, this has had a significant positive impact on global gross farm income. In 2024, this amounted to $29 billion, equivalent to having added 6.4% to the value of global production of the four main crops of soybeans, maize, canola and cotton. Since 1996, gross farm incomes have increased by $384.9 billion.

At the country level, US farmers have been the largest beneficiaries of higher incomes, realizing $165 billion in extra income between 1996 and 2024. This is not surprising given that US farmers were first to make widespread use of GM crop technology and for many years the GM adoption levels in all four US crops have been more than 85%. Important farm income benefits ($117.9 billion) have occurred in South America (Argentina, Bolivia, Brazil, Colombia, Paraguay and Uruguay), mostly from GM technology in soybeans and maize. GM IR cotton has also been responsible for an additional $66.5 billion additional income for cotton farmers in China and India.

In 2024, 56% of the farm income benefits were earned by farmers in developing countries. Most of these gains have been from GM IR cotton and GM HT soybeans. Over the period 1996–2024, the cumulative farm income gain derived by developing country farmers was $200.1 billion, equal to 52% of the total farm income during this period.

The average cost to farmers for accessing GM technology, across the four main crops, 1996–2024, was equal to 24% of the total value of technology gains. This is defined as the farm income gains referred to above plus the cost of the technology payable to the seed supply chain. Readers should note that the cost of the technology accrues to the seed supply chain including sellers of seed to farmers, seed multipliers, plant breeders, distributors and the GM technology providers.

In developing countries, the total cost was equal to 19% of total technology gains compared with 29% in developed countries. While circumstances vary between countries, the higher share of total technology gains accounted for by farm income in developing countries relative to developed countries reflects factors such as weaker provision and enforcement of intellectual property rights in developing countries and the higher average level of farm income gain per hectare derived by farmers in developing countries compared to those in developed countries.

In terms of investment, this means that for each extra dollar invested in GM crop seeds (relative to the cost of conventional seed), farmers gained an average US $4.17 in extra income. In developing countries, the average return was $5.31 for each extra dollar invested in GM crop seed and in developed countries the average return was $3.47.

Sixty-eight percent of the total income gain over the 29-y period derived from higher yields and second crop soybean gains with 32% from lower costs (mostly on insecticides and herbicides). In terms of the two main trait types, insect resistance and herbicide tolerance have accounted for 55.1% and 44.7% respectively of the total income gain (other traits of drought resistant maize and virus-resistant papaya and squash accounted for the 0.2% balance). The balance of the income gain arising from yield/production gains relative to cost savings has been changing as second- and third-generation GM crops have been increasingly adopted.

### Crop Production Impacts

The positive yield impacts identified above plus the second soybean crop facilitation in South America have delivered important volumes to the global production of maize, cotton, canola and soybeans since 1996 (Table 2). The GM IR traits, used in maize and cotton, have accounted for 90% of the additional maize production and 98.6% of the additional cotton production. The small residual production gains have come from improvements in levels of weed control.

**Table 2.** Additional crop production arising from positive yield effects of GM crops (million tonnes).

	1996–2024	2024
Soybeans	459.16	37.38
Maize	816.66	58.0
Cotton	45.64	2.13
Canola	21.01	1.24
Sugar beet	2.48	0.17

Note: Sugar beet, US and Canada only (from 2008).

In soybeans, the second cropping in South America, enabled by increased use of NT/RT production systems, facilitated by GM HT technology has added 277 million tonnes to global soybean production, with Intacta soybeans added a further 81.9 million tonnes since 2013. The remaining additional GM-related soybean production has come from the second generation of GM HT soybeans grown in North American countries since 2008 and where the GM HT technology has enabled farmers to obtain higher yield via improved levels of weed control.

## Concluding Comments

Since the mid-1990s, GM crop seed technology has helped many farmers to grow more food, feed and fiber using fewer resources by reducing the damage caused by pests and better controlling weeds. The highest yield increases have occurred in developing countries, and this has contributed to a more reliable and secure food supply base in these countries. In South America, HT technology has helped farmers adopt RT/NT production systems, shortening the time between planting and harvesting, allowing them the opportunity to grow an additional soybean crop after wheat in the same growing season.

With higher yields and less time and money spent managing pests and weeds, farmers have earned higher incomes. This has proven to be especially valuable for farmers in developing countries where, over the period 1996–2024, an average $5.31 was received for each extra dollar invested in biotech crop seeds.

The widespread use of GM crop technology has also contributed to changing agriculture’s land footprint by allowing farmers to grow more on existing land used for agricultural purposes, reducing the pressure to bring in new land into cultivation. For example, if world agriculture wanted to maintain global production of the four main crops in which GM seed technology has been widely used levels at 2024 levels, but without using the GM technology, this would require farmers to plant an additional 12.8 million ha of soybeans, 9.6 million ha of maize, 2.5 million ha of cotton and 0.6 million ha of canola, an area (25.5 million ha in total) equivalent to the combined agricultural area of the Philippines and Vietnam.

Nevertheless, in relation to the use of HT crops, over reliance on the use of glyphosate and the lack of crop and herbicide rotation by farmers, in some regions, has contributed to the development of weed resistance. In order to address this problem and maintain good levels of weed control, farmers have increasingly adopted more integrated weed management strategies incorporating a mix of herbicides, other HT crops and cultural weed control measures (in other words using other herbicides with glyphosate rather than solely relying on glyphosate, using HT crops which are tolerant to other herbicides, such as dicamba, 2,4-D and glufosinate and using cultural practices such as mulching and reverting to plowing). This has added cost to the GM HT production systems relative to the costs of the early years of adoption in the late 1990s. Despite this, relative to the current conventional alternative, the GM HT technology continues to offer important economic benefits in 2024, as evidenced by the continued widespread use of this technology.

Similarly, in relation to GM IR crops there have been some incidence of pest resistance to IR traits occurring. This led to growers in regions affected adjusting their pest control strategies to include measures such as supplementing the use of GM IR traited seed with other forms of pest control (e.g., use of some insecticides or bio-pesticides) and/or the adoption of newer, more expensive second and third generation IR traited seed with multiple modes of action to control pests rather than the single mode of actions more commonplace in the first generation of IR-traited seed. These changes aim to reduce the risk or slow down pest resistance development but have resulted in the overall pest control costs associated with growing GM IR crops generally being higher in the last 10 y than in the early years of adoption. Despite this, as with GM HT crop technology, the GM IR technology use continues to be popular with farmers because it continues to provide economic advantages for most users relative to the conventional (non-GM) alternative form of production.

The findings of this research with other research are consistent with the analysis of other global assessments of this technology by other authors such as the meta (global) analyses of Klumper and Qaim^17^ Finger R et al.^18^ and Areal F et al.^19^

## Statistical Sources

AMIS Global/Kleffmann. Subscription-based data sources (derived from farmer surveys) on pesticide use. Kleffmann GmbH, Ludinghausen, Germany. www.kleffmann.com

## Appendix A. Details of methodology as applied to 2020 farm income calculations

**Table A1.** GM IR corn (targeting corn boring pests) 2024.

Country	Area of trait (‘000 ha)	Yield assumption % change	Base yield (tonnes/ha)	Farm level price ($/tonne)	Cost of technology ($/ha)	Impact on costs, net of cost of technology ($/ha)	Change in farm income ($/ha)	Change in farm income at national level (‘000 $)	Production impact (‘000 tonnes)
US	28,367	+7	10.86	168	+24.51	+22.66	+105.13	+3,673,452	+25,733
Canada	1,286	+7	9.88	158	+21.6	+19.16	+90.10	+115,876	+899
Argentina	6,176	+5.5	7.57	155	+20.5	+20.5	+44.08	+272,227	+2,571
Philippines	709	+18	3.11	387	+34.91	+23.55	+193.28	+137,032	+397
South Africa	1,803	+10.6	5.03	278	+12.0	−1.09	+149.45	+269,422	+961
Spain	69	+12.6	11.25	245	+39.5	+32.56	+272.90	+18,939	+98
Uruguay	149	+5.5	5.74	157	+20.46	+20.46	+29.12	+4,331	+47
Honduras	67	+24	5.29	390	+100.0	+100.0	+94.59	+26,438	+85
Portugal	1	+12.5	10.78	187	+40.58	+40.58	+211.56	+192	+1
Brazil	20,963	+11.1	5.15	135	+27.27	+8.07	+68.80	+1,393,203	+11,566
Colombia	133	+16	5.16	410	+47.60	+5.80	+331.89	+44,114	+110
Paraguay	855	+5.5	5.49	130	+10.07	+10.07	+31.67	+27,076	+258
Vietnam	425	+10.2	4.81	270	+17.01	−16.44	+149.17	+63,395	+209
China	1,000	+7.65	6.69	464	+9.75	−11.4	+249.16	+249,156	+512

1. Impact on costs net of cost of technology = cost savings from reductions in pesticide costs, labor use, fuel use, etc., from which the additional cost (premium) of the technology has been deducted. For example (above) US cost savings from reduced expenditure on insecticides = -$15.9/ha, limited to an area equivalent to 10% of the total crop area (the area historically treated with insecticides for corn boring pests). This converted to an average insecticide cost saving equivalent per hectare of GM IR crop of -$1.85/ha. After deduction of the cost of technology (+$24.51/ha) is deducted to leave a net impact on costs of +$22.66 2. There are no Canadian-specific studies available, hence application of US study findings to the Canadian context (US being the nearest country for which relevant data is available).

**Table A2.** GM IR Corn (Targeting Corn Rootworm) 2024.

Country	Area of trait (‘000 ha)	Yield assumption % change	Base yield (tonnes/ha)	Farm level price ($/tonne)	Cost of technology ($/ha)	Impact on costs, net of cost of technology ($/ha)	Change in farm income ($/ha)	Change in farm income at national level (‘000 $)	Production impact (‘000 tonnes)
US	10,376	+5	10.86	168	+21.51	+21.15	+112.43	+1,166,503	+5,635
Canada	999	+5	9.88	158	+22	+3.99	+82.03	+82,020	+494

1. There are no Canadian-specific studies available, hence application of US study findings to the Canadian context (US being the nearest country for which relevant data is available).

**Table A3.** GM IR Cotton 2024.

Country	Area of trait (‘000 ha)	Yield assumption % change	Base yield (tonnes/ha)	Farm level price ($/tonne)	Cost of technology ($/ha)	Impact on costs, net of cost of technology ($/ha)	Change in farm income ($/ha)	Change in farm income at national level (‘000 $)	Production impact (‘000 tonnes)
US	3,141	+10	0.812	1,667	+71.28	+39.47	+95.89	+301,195	+255
China	2,707	+10	1.94	2,584	+50.95	−24.07	+525.37	+1,422,449	+525
South Africa	17	+24	0.69	2,832	+18.82	−11.89	+259.78	+4,442	+3
Australia	522	Zero	1.98	2,352	+211.22	−153.95	+153.95	+80,437	Zero
Mexico	87	+10.3	1.34	1,730	+60.71	−42.69	+95.34	+16,951	+12
Argentina	519	+30	0.456	1,804	+21.25	−32.36	+142.31	+73,914	+71
India	10,920	+24	0.359	1,473	+9.6	+12.6	+139.51	+1,523,399	+941
Colombia	7	+20.7	0.89	1,370	+73.10	+13.17	+60.71	+2,673	+1
Brazil	1,750	+2.4	1.81	1,705	+26.47	−8.16	+81.63	+142,837	+75
Pakistan	1,900	+10	0.566	2,556	+5.32	−0.09	+144.79	+275,094	+107
Myanmar	171	+30	0.515	2,556	+20	+14.59	+380.39	+65,046	+26

Note Myanmar price based on Pakistan.

**Table A4.** GM HT Soybeans 2024 (Excluding Second Crop Soybeans – See Separate Table).

Country	Area of trait (‘000 ha)	Yield assumption % change	Base yield (tonnes/ha)	Farm level price ($/tonne)	Cost of technology ($/ha)	Impact on costs, net of cost of technology ($/ha)	Change in farm income ($/ha)	Change in farm income at national level (‘000 $)	Production impact (‘000 tonnes)
US 1^st^generation	1,341	Nil	3.41	362	+9.68	−46.65	+46.65	+62,537	Nil
US 2^nd^ generation	32,173	+8.9	3.17	362	+24.82	−31.49	+133.5	+4,294,944	+9,077
Canada 1^st^ generation	11	Nil	3.13	353	+10.0	−77.15	−77.15	+829	Nil
Canada 2^nd^ generation	1,840	+8.9	2.92	353	+37.29	−49.86	+141.58	+260,499	+478
Argentina	11,796	Nil	2.84	301	+2.5	−23.83	+23.83	+281,116	Nil
Brazil	2,797	Nil	3.62	496	+21.42	−59.28	+59.28	+165,793	Nil
Paraguay	1,464	Nil	2.91	391	+4.4	−20.66	+20.66	+30,248	Nil
South Africa	1,140	Nil	2.41	424	+1.13	−3.09	+3.09	+3,526	Nil
Uruguay	883	Nil	2.38	570	+2.5	−31.14	+31.14	+27,488	Nil
Bolivia	1,562	+15	1.72	356	+3.32	−5.96	+80.84	+126,250	+403

Notes.

1. Price discount for GM soybeans relative to non GM soybeans in Bolivia of 2.7% - price for non GM soybeans was $366/tonne - price shown above is discounted.

**Table A5.** GM IR/HT (Intacta) Soybeans 2024.

Country	Area of trait (000’ ha)	Yield assumption % change	Base yield sucrose(tonnes/ha)	Farm level price: $/tonne)	Cost of tech ($/ha)	Impact on costs, net of cost of tech ($/ha)	Change in farm income ($/ha)	Change in farm income at national level (‘000 $)	Production impact (‘000 tonnes)
Brazil	44,177	+9.4	3.23	496	+48.51	+3.67	+146.9	+6,489,404	+8,624
Argentina	5,070	+7.1	2.96	301	+49.75	+8.82	+54.43	+275,979	+1,064
Paraguay	2,378	+11.5	2.82	391	+48.83	−13.3	+140.11	+333,225	+771
Uruguay	378	+7	2.33	570	+49.75	+0.3	+92.93	+35,156	+62

**Table A6.** GM HT Corn 2024.

Country	Area of trait (‘000 ha)	Yield assumption % change	Base yield (tonnes/ha)	Farm level price ($/tonne)	Cost of technology ($/ha)	Impact on costs, net of cost of technology ($/ha)	Change in farm income ($/ha)	Change in farm income at national level (‘000 $)	Production impact (‘000 tonnes)
US	30,123	Nil	11.52	168	+25.25	−40.51	+40.51	+1,220,313	Nil
Canada	1,315	Nil	10.48	158	+21.51	−23.60	+23.60	+31,052	Nil
Argentina: as single trait	160	+3% con belt, +22% marginal areas	8.64 corn belt, 5.36 marginal areas	155	+17.25	−1.6	+40.18 corn belt, +182.78 marginal areas	+19,862	+130
Argentina: as stacked trait	6,176	+10.25	7.57	155	+1.3	−14.4	+134.67	+831,699	+4,792
South Africa	1,980	Nil	5.03	278	+9.49	−3.56	+3.56	+7,042	Nil
Philippines	709	+5	3.11	387	+34.19	+12.31	+147.91	+33,971	+110
Colombia	148	Zero	5.66	410	+16.8	−7.12	+7.12	+1,052	Nil
Brazil	20,502	+3	5.15	135	+17.25	+10.09	+12.72	−260,747	+3,027
Uruguay	153	Nil	5.88	157	+15.39	+5.3	+5.3	+804	Nil
Paraguay	855	Nil	5.78	138	+17.25	+2.7	+2.7	+2,311	Nil
Vietnam	425	+5	4.81	270	+8.38	+25.59	+90.53	+38,475	+102
China	1,000	Nil	6.7	464	+3.25	−5.14	+5.14	5,140	Nil

1. Where no positive yield effect due to this technology is applied, the base yields shown are the indicative average yields for the crops and differ (are higher) than those used for the GM IR base yield analysis, which have been adjusted downwards to reflect the impact of the yield enhancing technology (see below) 2. Argentina: single trait. In the Corn Belt it is assumed that 70% of trait plantings occur in this region and marginal regions account for the balance. In relation to stacked traits, the yield impact (+10.25%) is in addition to the yield 5.5% impact presented for the GM IR trait (above). In other words the total estimated yield impact of stacked traits is +15.75%. The cost of the technology also relates specifically to the HT part of the technology (sold within the stack).

**Table A7.** GM HT Cotton 2024.

Country	Area of trait (‘000 ha)	Yield assumption % change	Base yield (tonnes/ha)	Farm level price ($/tonne)	Cost of technology ($/ha)	Impact on costs, net of cost of technology ($/ha)	Change in farm income ($/ha)	Change in farm income at national level (‘000 $)	Production impact (‘000 tonnes)
US	3,246	Nil	0.885	1,667	+47.52	−72.53	+72.53	+235,412	Nil
S Africa	18	Nil	0.85	1,390	+11.8	−7.18	+7.18	+129	Nil
Australia	550	Nil	1.98	2,352	−56.11	−22.89	+22.89	+12,592	Nil
Argentina	530	Farm saved seed area nil Certified seed area +9.3%	0.589	473	+11.76 certified seed, nil farm saved seed	−5.84 certified seed, −17.6 farm saved seed	+84.98 certified seed, +17.6 farm saved seed	+14,459	+9
Mexico	122	+16	1.34	1,730	+45.1	−27.19	+342.57	+41,870	+26
Colombia	7	+4.0	0.895	2,022	+34.2	−29.47	+101.87	+955	+0.3
Brazil	1,944	+1.6	1.81	1,705	+8.93	−12.1	+80.73	+156,948	+78

1. Where no positive yield effect due to this technology is applied, the base yields shown are the indicative average yields for the crops and differ (are higher) than those used for the GM IR base yield analysis, which have been adjusted downwards to reflect the impact of the yield enhancing technology (see below). 2. Argentina: 30% of area assumed to use certified seed with 70% farm saved seed.

**Table A8.** GM HT Canola 2024.

Country	Area of trait (‘000 ha)	Yield assumption % change	Base yield (tonnes/ha)	Farm level price ($/tonne)	Cost of technology ($/ha)	Impact on costs, net of cost of technology ($/ha)	Change in farm income ($/ha)	Change in farm income at national level (‘000 $)	Production impact (‘000 tonnes)
US glyphosate tolerant	224	+3.46	1.92	448	+17.3	−5.22	+34.95	+7,843	+17
US glufosinate tolerant	874	+6.63	1.92	448	+17.3	+9.82	+47.18	+41,257	+92
Canada glyphosate tolerant	1,725	+4.6	2.14	467	+5.87	−22.52	+72.24	+124,597	+169
Canada glufosinate tolerant	6,688	+6.5	2.14	467	+16.59	−7.48	+65.78	+439,918	+930
Australia glyphosate tolerant	800	+8	1.69	480	+10.36	+2.64	+46.32	+37,053	+77

Note: Baseline (conventional) comparison in Canada with herbicide tolerant (non-GM) “Clearfield” varieties.

**Table A9.** GM Virus Resistant Crops 2024.

Country	Area of trait (ha)	Yield assumption % change	Base yield (tonnes/ha)	Farm level price ($/tonne)	Cost of technology ($/ha)	Impact on costs, net of cost of technology ($/ha)	Change in farm income ($/ha)	Change in farm income at national level (‘000 $)	Production impact (‘000 tonnes)
US Papaya	187	+17	12.21	968	+494	+494	+1,515	+283	+0.4
US squash	1,000	+100	19.6	575	+736	+736	+10,536	+10,536	+20

**Table A10.** GM Herbicide Tolerant Sugar Beet 2024.

Country	Area of trait (000’ ha)	Yield assumption % change	Base yield sucrose(tonnes/ha)	Farm level price equivalent (sucrose: $/tonne)	Cost of tech ($/ha)	Impact on costs, net of cost of tech ($/ha)	Change in farm income ($/ha)	Change in farm income at national level (‘000 $)	Production impact (‘000 tonnes)
US	440	+3.38	10.78	478	+148	−43.2	+217.33	+95,533	+200
Canada	19	+3.38	16.56	478	+148	−43.2	+310.69	+5,841	+12

**Table A11.** GM Drought Tolerant Maize 2024.

Country	Area of trait (000’ ha)	Yield assumption % change	Base yield (tonnes/ha)	Farm level price: $/tonne	Cost of tech ($/ha)	Impact on costs, net of cost of tech ($/ha)	Change in farm income ($/ha)	Change in farm income at national level (‘000 $)	Production impact (‘000 tonnes)
US	1,042	+2.57	10.87	168	−19.21 (discount)	+19.28	+66.2	+68,959	+410

**Table A12.** GM IR Brinjal 2024.

Country	Area of trait (ha)	Yield assumption % change	Base yield (tonnes/ha)	Farm level price $/tonne	Cost of tech ($/ha)	Impact on costs, net of cost of tech ($/ha)	Change in farm income ($/ha)	Change in farm income at national level (‘000 $)	Production impact (‘000 tonnes)
Bangladesh	4,088	+19.6	9.89	217	Nil	−67.69	+681.13	+2,785	+14

### Second Soybean Crop Benefits: Argentina

An additional farm income benefit that many Argentine soybean growers have derived comes from the additional scope for second cropping of soybeans. This has arisen because of the simplicity, ease and weed management flexibility provided by the (GM) technology which has been an important factor facilitating the use of no and reduced tillage production systems. In turn, the adoption of low/no tillage production systems has reduced the time required for harvesting and drilling subsequent crops and hence has enabled many Argentine farmers to cultivate two crops (wheat followed by soybeans) in one season. As such, the proportion of soybean production in Argentina using no or low tillage methods has increased from 34% in 1996 to 90% by 2005 and has remained at over 90% since then.

**Table A13.** Farm Level Income Impact of Using GM HT Soybeans in Argentina 2024 (2): Second Crop Soybeans.

Year	Second crop area (million ha)	Average gross margin/ha for second crop soybeans ($/ha)	Increase in income linked to GM HT system (million $)
2024	5.4	238.4	1,288.9

Source & notes: 1. Crop area and gross margin data based on data supplied by Grupo CEO and the Argentine Ministry of Agriculture 2. The second cropping benefits are based on the gross margin derived from second crop soybeans multiplied by the total area of second crop soybeans.

### Base Yields Used Where GM Technology Delivers a Positive Yield Gain

To avoid over-stating the positive yield effect of GM technology (where studies have identified such an impact) when applied at a national level, average (national level) yields used have been adjusted downwards (see example below). Production levels based on these adjusted levels were then cross checked with total production values based on reported average yields across the total crop.

**Table A14.** Example: GM IR Cotton (2024).

Country	Average yield across all forms of production (t/ha)	Total cotton area (‘000 ha)	Total production (‘000 tonnes)	GM IR area (‘000 ha)	Conventional area (‘000 ha)	Assumed yield effect of GM IR technology	Adjusted base yield for conventional cotton (t/ha)	GM IR production (‘000 tonnes)	Conventional production (‘000 tonnes)
US	0.885	3,490	3,089	3,141	349	+10%	0.812	2,806	283
China	2.124	2,850	6,053	2,708	143	+10%	1.94	5,778	276

Note: Figures subject to rounding.

## Appendix B: Trait specific summaries of impacts and sources

**Table B1.** GM HT soybeans: summary of average gross farm level income impacts 1996–2024.

Country	Cost of technology ($/ha)	Average gross farm income benefit (after deduction of cost of technology: $/ha)	Aggregate income benefit (million $)	Type of benefit	References
*1*^*st*^ *generation GM HT soybeans*					
Romania (to 2006 only)	50–60	104	44.6	Small cost savings of about $9/ha, balance due to yield gains of +13% to +31%	Brookes^1^ Monsanto Romania^20^
Argentina	2–4	22.5 plus second crop benefits of 211	29,342.7	Cost savings plus second crop gains	Qaim and Traxler^21^ Trigo and CAP,^22^ Rodriguez et al.^23^ and updated from 2008 to reflect herbicide usage and price changes
Brazil	7–31	33.9	10,058.1	Cost savings	Parana Department of Agriculture^24^ Galveo^25^^,^^26^^,^^27^^,^^28^ and updated to reflect herbicide usage and price changes
US	10–57	33.5	14,436.8	Cost savings	Marra et al.^29^ Carpenter and Gianessi^30^ Sankala and Blumenthal^8^^,^^9^ Johnson and Strom^31^ And updated to reflect herbicide price and common product usage
Canada	10–48	21	241.13	Cost savings	George Morris Center^32^ and updated to reflect herbicide price and common product usage
Paraguay	4–10	16.6 plus second crop benefits of 311	1,522.6	Cost savings	Based on Argentina as no country-specific analysis identified. Impacts confirmed by industry sources and herbicide costs and usage updated 2009 onwards from herbicide survey data (AMIS Global/Kleffman/Kynetec)
Uruguay	2–4	23.9	368.1	Cost savings	Based on Argentina as no country-specific analysis identified. Impacts confirmed by industry sources and herbicide costs and usage updated 2009 onwards from herbicide survey data (AMIS Global/Kleffman/Kynetec)
South Africa	2–30	7.2	82.3	Cost savings	As there are no published studies available, based on data from industry sources and herbicide costs and usage updated 2009 onwards from herbicide survey data (AMIS Global/Kleffman/Kynetec)
Mexico	20–47	40	6.1	Cost savings plus yield impacts in range of −2% to +13%	Monsanto/Bayer annual monitoring reports submitted to Ministry of Agriculture and personal communications
Bolivia	3–4	83.2	1,696.1	Cost savings plus yield gain of +15%	Fernandez W et al.^33^
*2nd*^*t*^ *generation GM HT soybeans*					
US and Canada	13–67	133.3 (US) 123.4 (Can)	42,546.1 (US) 2,430.1 (Can)	Cost savings as first generation plus yield gains in range of +5% to +11%	As first-generation GM HT soybeans plus annual farm level survey data from Monsanto/Bayer USA
*Intacta soybeans*					
Brazil	29–53	122.4	34,807.4	Herbicide cost saving as 1^st^ generation plus insecticide saving $19/ha and yield gain +9% to +10%	Monsanto/Bayer Brazil pre commercial trials and post market (farm survey) monitoring, MB Agro,^34^ Horikoshi R et al.^35^
Argentina	19–53	66.8	2,543.5	Herbicide cost saving as 1^st^ generation plus insecticide saving $21/ha and yield gain +7% to +9%	Monsanto/Bayer Argentina pre commercial trials and post market monitoring surveys
Paraguay	29–53	132.2	2,262.8	Herbicide cost saving as 1^st^ generation plus insecticide saving $33/ha and yield gain +9% to +13%	Monsanto/Bayer Paraguay pre commercial trials and post market monitoring surveys
Uruguay	19–53	77	272.7	Herbicide cost saving as 1^st^ generation plus insecticide saving $19/ha and yield gain +7% to +9%	Monsanto/Bayer Uruguay pre commercial trials and post market monitoring surveys

Notes: 1. Romania stopped growing GM HT soybeans in 2007 after joining the European Union, where the trait is not approved for planting. Mexico has not planted any GM HT soybeans since 2017 because of a government ban its cultivation 2. The range in values for cost of technology relates to annual changes in the average cost paid by farmers. It varies for reasons such as the price of the technology set by seed companies, exchange rates, average seed rates and values identified in different studies 3. Intacta soybeans (HT and IR) first grown commercially in 2013 4. For additional details of how impacts have been estimated, see examples in Appendix 1 5. AMIS Global/Kleffmann/Kynetec are subscription-based data sources (derived from farmer surveys) on pesticide use 6. References to Monsanto/Bayer Argentina, Brazil, Paraguay and Uruguay as sources of data from pre-commericalisation trials and post market monitoring – this is unpublished data provided to the authors by these companies on a yearly basis covering seed premium, yield comparisons and cost of insecticide/number of insecticide treatment comparisons for Intacta crops versus conventional and GM HT (only) crops. The data derive from survey-based monitoring of sites growing each crop.

**Table B2.** GM HT maize: summary of average gross farm income impacts 1997–2024.

Country	Cost of technology ($/ha)	Average gross farm income benefit (after deduction of cost of technology: $/ha)	Aggregate income benefit (million $)	Type of benefit	References
US	15–29	32.8	17,701.8	Cost savings	Carpenter and Gianessi^30^ Sankala and Blumenthal^8^^,^^9^ Johnson and Strom^31^ Also updated annually to reflect herbicide price and common product usage
Canada	17–35	15.5	364.7	Cost savings	Monsanto/Bayer Canada (personal communications) and updated annually since 2008 to reflect changes in herbicide prices and usage
Argentina	2–20	120.7	8,717.9	Cost savings plus yield gains over 10% and higher in some regions	Personal communications from Monsanto/Bayer Argentina, Grupo CEO and updated since 2008 to reflect changes in herbicide prices and usage
South Africa	9–18	3.3	91.3	Cost savings	Personal communications from Monsanto/Bayer South Africa and updated since 2008 to reflect changes in herbicide prices and usage
Brazil	10–32	21.3	4,015.9	Cost savings plus yield gains of +1% to +7%	Galveo^25^^,^^26^^,^^27^^,^^28^
Colombia	14–24	11.1	15.1	Cost savings	Mendez et al.,^36^ Brookes^37^
Philippines	24–47	31.7	344.6	Cost savings plus yield gains of +5% to +15%	Gonsales^38^ Monsanto/Bayer Philippines (personal communications) Updated since 2010 to reflect changes in herbicide prices and usage
Paraguay	11–17	2.6	17.1	Cost saving	Personal communications from Monsanto/Bayer Paraguay and AMIS Global/Kleffman/Kynetec – annually updated to reflect changes in herbicide prices and usage
Uruguay	6–17	0.2	0.4	Cost saving	Personal communication from Monsanto/Bayer Uruguay and AMIS Global/Kleffman/Kynetec - updated annually to reflect changes in herbicide prices and usage
Vietnam	11–28	88.2	122.7		Brookes,^39^ Brookes and Dinh^40^
China	3.25	4.9	5.7	Cost saving	Quan Y et al.^11^

1. The range in values for cost of technology relates to annual changes in the average cost paid by farmers. It varies for reasons such as the price of the technology set by seed companies, exchange rates, average seed rates and values identified in different studies 2. For additional details of how impacts have been estimated, see examples in Appendix 1 3. AMIS Global/Kleffmann/Kynetec are subscription-based data sources (derived from farmer surveys) on pesticide use 4. References to Monsanto Bayer Argentina, Canada, South Africa, Philippines, Paraguay and Uruguay as sources of data – this is unpublished data provided to the authors by these companies on a yearly basis covering seed premium and typical herbicide treatments used on GM HT and conventional crops 5. Reference to changes in herbicide prices and usage – author estimates drawing on AMIS Global/Kleffmann/Kynetec data and other similar database sources and extension services (e.g., Ontario Ministry of Agriculture in Canada).

**Table B3.** GM HT Cotton Summary of Average Gross Farm Income Impacts 1997–2024.

Country	Cost of technology ($/ha)	Average gross farm income benefit (after deduction of cost of technology: $/ha)	Aggregate income benefit (million $)	Type of benefit	References
US	13–82	25.2	2,131.6	Cost savings	Carpenter and Gianessi^30^ Sankala and Blumenthal^8^^,^^9^ Johnson and Strom^31^ Also updated to reflect herbicide price and common product usage
South Africa	11–32	25.8	8.6	Cost savings	Personal communications from Monsanto/Bayer South Africa and updated since 2008 to reflect changes in herbicide prices and usage
Australia	32–82	23.5	176.2	Cost savings	Doyle et al.^41^ Monsanto/Bayer Australia (personal communications) and updated to reflect changes in herbicide usage and prices
Argentina	10–30	37.8	299.5	Cost savings and yield gain of +9%	Personal communications from Monsanto/Bayer Argentina, Grupo CEO and updated since 2008 to reflect changes in herbicide prices and usage
Brazil	8–53	70.2	1,010.0	Cost savings plus yield gains of +1.6% to +4%	Galveo^25^^,^^26^^,^^27^^,^^28^
Mexico	29–79	331.9	807.6	Cost savings plus yield gains of +3% to +20%	Monsanto/Bayer Mexico annual monitoring reports submitted to the Ministry of Agriculture and personal communications
Colombia	34–96	67.4	22.3	Cost savings plus yield gains of +4% (note −5% in first year of adoption − 2008/09)	Monsanto/Bayer Colombia annual personal communications, Brookes^37^

1. The range in values for cost of technology relates to annual changes in the average cost paid by farmers. It varies for reasons such as the price of the technology set by seed companies, exchange rates, average seed rates, the nature and effectiveness of the technology (e.g., second generation ‘Flex’ cotton offered more flexible and cost-effective weed control than the earlier first generation of HT technology) and values identified in different studies 2. For additional details of how impacts have been estimated, see examples in Appendix 1 3. Note negative yield impact of yield in first year of adoption mainly due to technology not being available in leading and locally adapted varieties 4. References to Monsanto/Bayer Argentina, Australia, South Africa and Colombia as sources of data – this is unpublished data provided to the authors by these companies on a yearly basis covering seed premium and typical herbicide treatments used on GM HT and conventional crops 5. Reference to Monsanto/Bayer Mexico annual monitoring reports. These are unpublished, annual monitoring of crop reports that the company is required to submit to the Mexican Ministry of Agriculture, as part of post market monitoring requirements. This provides data on seed premia, cost of weed control and production and yields for GM HT cotton versus conventional to a regional level 6. Reference to changes in herbicide prices and usage – author estimates drawing on AMIS Global/Kleffmann/Kynetec data and other similar database sources and extension services (e.g., New South Wales Department of Agriculture in Australia).

**Table B4.** Other GM HT Crops Summary of Average Gross Farm Income Impacts 1996–2024.

Country	Cost of technology ($/ha)	Average farm income benefit (after deduction of cost of technology: $/ha)	Aggregate income benefit (million $)	Type of benefit	References
*GM HT canola*					
US	12–33	45.9	648.2	Mostly yield gains of +1% to +12% (especially Invigor canola)	Sankala and Blumenthal^8^^,^^9^ Johnson and Strom^31^ And updated to reflect herbicide price and common product usage
Canada	4–34	64.2	10,590.5	Mostly yield gains of +3% to +12% (especially Invigor canola)	Canola Council^42^ Gusta et al.^43^ and updated to reflect herbicide price changes and seed variety trial data (on yields)
Australia	9–41	47.9	311.5	Mostly yield gains of +12% to +22% (where replacing triazine tolerant canola) but no yield gain relative to other non-GM (herbicide tolerant canola)	Monsanto Australia,^44^ Fischler and Tozer^45^ and Hudson and Richards^46^
*GM HT sugar beet*					
US and Canada	130–151	146	1,119.7	Mostly yield gains of +3% to +13%	Kniss^47^ Khan^48^ Jon-Joseph et al.^49^ Annual updates of herbicide price and usage data

Notes.

1. In Australia, one of the most popular type of production has been canola tolerant to the triazine group of herbicides (tolerance derived from non GM techniques). It is relative to this form of canola that the main farm income benefits of GM HT (to glyphosate) canola have occurred 2. InVigor’ hybrid vigor canola (tolerant to the herbicide glufosinate) is higher yielding than conventional or other GM HT canola and derives this additional vigor from GM techniques 3. The range in values for cost of technology relates to annual changes in the average cost paid by farmers. It varies for reasons such as the price of the technology set by seed companies, exchange rates, average seed rates and values identified in different studies 4. For additional details of how impacts have been estimated, see examples in Appendix 1 5. References to Monsanto Australia as a source of data – this is unpublished data provided to the authors by this company on a yearly basis covering seed premium and typical herbicide treatments used on GM HT and conventional crops 6. Reference to changes in herbicide prices and usage – author estimates drawing on AMIS Global/Kleffmann/Kynetec data.

**Table B5.** Average (%) Yield Gains GM IR Cotton and Maize 1996–2024.

	Maize insect resistance to corn boring pests	Maize insect resistance to rootworm pests	Cotton insect resistance	References
US	7.0	5.0	9.9	Carpenter and Gianessi^30^ Marra et al.^29^ Sankala and Blumenthal^8^^,^^9^ Hutchison et al.^50^ Rice^51^ Mullins and Hudson^10^
China	7.2	N/a	10.0	Pray et al.^52^
South Africa	10.9	N/a	24.0	Gouse et al.^53^^,^^54^^,^^55^ Van der Wald^56^ Ismael et al.^57^ Kirsten et al.^58^ James^59^
Honduras	24	N/a	N/a	Falk Zepeda et al.^60^^,^^61^
Mexico	N/a	N/a	11.0	Traxler and Godoy-Avila S^6^ Monsanto Mexico annual cotton monitoring reports^7^
Argentina	5.7	N/a	30.0	Trigo^62^ Trigo and Cap^22^ Rodriguez et al.^23^ Qaim and De Janvry^63^^,^^64^ Elena^65^
Philippines	18.1	N/a	N/a	Gonsales^66^ Gonsales et al.^38^ Yorobe^67^ Ramon^68^
Spain	11.7	N/a	N/a	Brookes^69^^,^^70^ Gomez-Barbero, Barbel M A and Rodriguez-Corejo^71^ Riesgo et al.^72^
Uruguay	5.5	N/a	N/a	As Argentina (no country-specific studies available and industry sources estimate similar impacts as in Argentina)
India	N/a	N/a	27.0	Bennett et al.^3^ IMRB^4^^,^^5^ Herring and Rao^73^
Colombia	16.7	N/a	25.0	Mendez et al.^36^ Zambrano et al.^74^ Brookes^37^
Canada	7.0	5.0	N/a	As US (no country-specific studies available and industry sources estimate similar impacts as in the US)
Burkina Faso	N/a	N/a	18.0	Vitale J et al.,^75^ Vitale J^76^
Brazil	11.3	N/a	2.1	Galveo^77^^,^^25^^,^^26^^,^^27^^,^^28^ Monsanto Brazil^78^
Pakistan	N/a	N/a	17.0	Nazli et al.,^79^ Kouser and Qaim^80^^,^^81^
Myanmar	N/a	N/a	30.4	USDA^82^
Australia	N/a	N/a	Nil	Doyle^83^ James^84^ CSIRO^85^ Fitt^86^
Paraguay	5.5	N/a	Not available	As Argentina (no country-specific studies available and industry sources estimate similar impacts as in Argentina)
Vietnam	9.9	N/a	N/a	Brookes,^39^ Brookes and Dinh^40^

Notes.

1. N/a = not applicable 2. Not included in table – also IR brinjal grown in Bangladesh an average yield gain 2013/14 to 2018/19 of +17.3% 3. 7% yield gain in the US associated with performance of GM IR technology to suppress corn boring pests includes benefits to non GM maize growers via areawide suppression of pests, estimated by Hutchison et al (202044) to account for 60% of the gross gains in the US between 1996 and 2009 4. Reference to Monsanto/Bayer Mexico annual monitoring reports. These are unpublished, annual monitoring of crop reports that the company is required to submit to the Mexican Ministry of Agriculture, as part of post market monitoring requirements. This provides data on seed premia, cost of pest control and production and yields for GM IR cotton versus conventional to a regional level 5. GM IR maize performance in Uruguay and Paraguay. Industry sources consulted for using Argentina impact data as a suitable proxy for impact in these countries include Monsanto/Bayer Argentina, Uruguay and Paraguay, Argenbio (Argentine Biotechnology Association) and Trigo E (Grupo CEO).

**Table B6.** GM IR crops: average gross farm income benefit 1996–2024.

Country	GM IR maize: cost of technology: $/ha	GM IR maize (income benefit after deduction of cost of technology: $/ha)	Aggregate income benefit GM IR maize (million $)	GM IR cotton: cost of technology: $/ha	GM IR cotton (income benefit after deduction of cost of technology: $/ha)	Aggregate income benefit GM IR cotton (million $)
US	17–32 IRCB, 20–42 IR CRW	101 IRCB, 87 IR CRW	76,6920.2	26–71	116	7,069.1
Canada	17–26 IRCB, 22–42 IR CRW	75 IRCB 89 IR CRW	3,028.0	N/a	N/a	N/a
Argentina	10–33	35	3,058.6	21–86	211	1,244.6
Philippines	30–47	119	1,293.6	N/a	N/a	N/a
South Africa	9–17	99	3,337.7	14–50	234	74.9
Spain	17–51	225	393.7	N/a	N/a	N/a
Uruguay	11–33	30	57.5	N/a	N/a	N/a
Honduras	100	170	100.5	N/a	N/a	N/a
Colombia	30–49	278	365.7	73–112	299	100.0
Brazil	44–69	64	14,646.1	25–52	96	435.1
China	9.75	236	N/a	38–60	399	26,268.6
Australia	N/a	N/a	N/a	85–299	193	1,135.6
Mexico	N/a	N/a	N/a	48–75	217	407.4
India	N/a	N/a	N/a	11–54	175	27,370.4
Burkina Faso	N/a	N/a	N/a	51–54	97	204.6
Myanmar	N/a	N/a	N/a	17–20	217	552.8
Pakistan	N/a	N/a	N/a	5–15	178	5,531.3
Paraguay	10–20	10	68.8	N/a	N/a	N/a
Vietnam	17–42	144	200.8		N/a	
**Average across alluser countries**		**85**			**206**	

Notes: 1. GM IR maize all are IRCB unless stated (IRCB = insect resistance to corn boring pests), IRCRW = insect resistance to corn rootworm 2. The range in values for cost of technology relates to annual changes in the average cost paid by farmers. It varies for reasons such as the price of the technology set by seed companies, the nature and effectiveness of the technology (e.g., second generation ‘Bollgard’ cotton offered protection against a wider range of pests than the earlier first generation of ‘Bollgard’ technology), exchange rates, average seed rates and values identified in different studies 3. Average across all countries is a weighted average based on areas planted in each user country 4. n/a = not applicable 5. Sources – as above yields table.
